# Whole-body vibration decreases pain and cartilage degeneration in male and female mice with osteoarthritis

**DOI:** 10.1097/PR9.0000000000001442

**Published:** 2026-04-28

**Authors:** Julia Temp, Melih Özgür Celik, Urszula Zabarylo, Dominika Labuz, Kay Raum, Stefan Zachow, Halina Machelska

**Affiliations:** a Department of Experimental Anesthesiology, Charité—Universitätsmedizin Berlin, Berlin, Germany; b Center for Biomedicine, Charité—Universitätsmedizin Berlin, Berlin, Germany; c Department of Mathematics for Life and Materials Sciences, Research Group on Computational Diagnosis and Therapy Planning, Zuse Institute Berlin, Berlin, Germany; d Department of Urology, Eberhard Karls University of Tuebingen, Tuebingen, Germany, Faculty of Medicine, Danube Private University, 3500 Krems an der Donau, Austria

**Keywords:** Cartilage, Microcomputed tomography, Nonpharmacological therapy, Osteoarthritis, Pain behavior, Whole-body vibration

## Abstract

**Introduction:**

Knee osteoarthritis (OA) is a chronic, degenerative joint disorder characterized by cartilage deterioration and persistent pain. Currently, there are no disease-modifying drugs and pharmacological treatments are unsatisfactory for OA.

**Objective:**

To investigate the therapeutic effects of whole-body vibration (WBV), a nondemanding physical intervention, on pain behaviors and cartilage morphology in male and female mice with knee OA.

**Methods:**

A medial meniscal transection (MMT) was used as a model that mimics meniscal tear–induced knee OA in patients. Animals were exposed to WBV (15-Hz frequency, 332-µm amplitude, 0.3*g* acceleration) for 15 minutes per day, 5 days per week, for 13 weeks, beginning 3 weeks after MMT, using a custom-made WBV device. Behavioral analysis included assessment of mechanical sensitivity (by von Frey filaments), the hind limb use (by dynamic weight-bearing), heat sensitivity (by Hargreaves test), locomotor activity (in open field boxes), and body weight. Microcomputed tomography was used to examine cartilage morphology.

**Results:**

Medial meniscal transection triggered mechanical hypersensitivity, reduced OA limb use, and induced cartilage damage in the medial and lateral compartments of femur and tibia, in both sexes. Whole-body vibration substantially alleviated mechanical hypersensitivity and improved limb use for up to 4 months after MMT, without affecting heat sensitivity, locomotor activity, or body weight. Furthermore, WBV considerably reduced the percentage of subchondral bone denuded of cartilage and increased cartilage thickness in the femur and tibia.

**Conclusion:**

These findings highlight the therapeutic potential of WBV as a nonpharmacological approach to relieve pain and restore cartilage integrity in knee OA.

## Abstract 2

Supplemental Digital Content is Available in the Text.

Whole-body vibration diminished mechanical hypersensitivity, restored limb use, and reduced cartilage damage in male and female mice with knee osteoarthritis, supporting its therapeutic potential.

## 1. Introduction

Knee osteoarthritis (OA) is a widespread, incurable degenerative disease affecting millions of people. It is the leading cause of disability and chronic pain, imposing personal and economic burdens.^12,29,38^ Risk factors include genetic predisposition, obesity, aging, trauma, a sedentary lifestyle, or excessive physical activity, with higher prevalence in women.^4,12,41^

Knee OA is characterized by the damage of articular cartilage. Healthy cartilage is avascular, aneural, and primarily composed of water, chondrocytes, and an extracellular matrix (EM) rich in collagen and proteoglycans. It covers the ends of knee bones (distal femur and proximal tibia) and distributes mechanical loads to prevent bone damage during movement. Joint trauma (eg, meniscal tear) increases metabolic activity of chondrocytes and causes cartilage fibrillation with microscopic cracks. As OA advances, EM erosion produces deep fissures, delamination, calcification, thinning, and loss of the cartilage. This exposes (denudes) the subchondral bone triggering its remodeling characterized by increased porosity, thickness, and osteophyte formation.^8,13^

Pain is a serious consequence of the OA joint pathology and it results from enhanced sensory neuron activation. The knee joint is richly innervated by peripheral sensory neuron fibers abundant in the capsule, ligaments, synovium, periosteum, subchondral bone, and menisci. During OA, the innervation of these tissues increases and activation of sensory fibers by proinflammatory and pronociceptive mediators (cytokines, neurotrophins, neuropeptides) secreted by chondrocytes, synovial immune cells, and neurons leads to peripheral sensitization. This process is followed by central sensitization due to sensory neuron and immune cell activation in the spinal cord and brain.^9,25^

Currently, there are no OA-modifying medications.^3^ Recommended interventions preceding pharmacological treatment are weight loss and physical activity.^4,25^ Whole-body vibration (WBV) is an emerging, physically nondemanding therapy, particularly suitable for patients with OA who usually are older and have attenuated muscle strength and impaired mobility.^32,35^ While industrial vibration can be detrimental,^17^ the controlled low-intensity (ie, low amplitude and low acceleration) therapeutic WBV can have beneficial effects on the musculoskeletal system. In preclinical models, WBV enhanced bone and muscle volume in healthy animals and in the brittle bone disease model,^44,49^ prevented intervertebral disc degradation induced by hind limb unloading,^14^ and improved recovery from the spinal cord injury.^48^ Notably, in patients with knee OA, WBV combined with quadriceps resistance exercise (squats, strengthening, stretching) diminished pain compared with those who did not exercise or only performed resistance exercise.^2,31,39,46^ However, the effects of sole WBV on OA pain and joint pathology remain unclear.

Given that cartilage damage and pain are debilitating consequences of OA, and disease severity may be sex-dependent,^13,29,41^ we comprehensively investigated the effects of WBV on cartilage and pain behaviors in male and female mice, using the medial meniscal transection (MMT) model, which resembles meniscal tear in patients with OA.^1,10^ Specifically, we evaluated the impact of WBV on mechanical and heat sensitivity, hind limb use, locomotion, body weight, and the cartilage morphology, for up to 4 months after MMT in both sexes.

## 2. Methods

Detailed methods are described in the supplemental digital content (http://links.lww.com/PR9/A403).

### 2.1. Animals

Experiments were approved by the State animal care committee and followed the ARRIVE guidelines.^20^ Adult male and female C57BL/6J mice (Janvier Laboratories) were kept in groups, with free access to food and water, in environmentally controlled conditions.^43^ Animals were randomly assigned to cages, and experimenters were blinded to the experimental conditions. Based on the sample size estimation (G*Power 3.1.2), 8 to 10 animals per group/sex were used.^43^ Animals were killed with isoflurane overdose. All efforts were made to minimize animal numbers and suffering.

### 2.2. Induction of osteoarthritis

Medial meniscal transection was performed under anesthesia on the right knee. The medial knee skin was shaved, disinfected, and incised with underlying muscles. The medial collateral ligament and the medial meniscus at its mid-body region were transected. The skin was closed with sutures. Sham surgery was performed analogously, except that ligament and meniscus were not transected.^43^ Naïve mice, without any surgery, were used as additional controls.

### 2.3. Whole-body vibration

**Figure 1.**
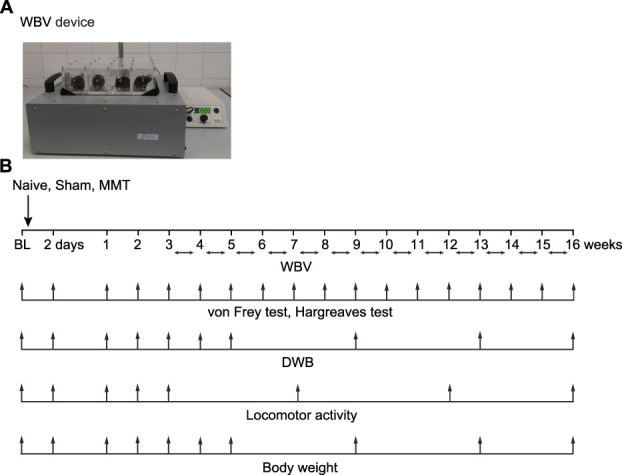
WBV experimental design. (A) The WBV device. The device consists of the vertically vibrating platform with 12 Plexiglas cages. The frequency of 15 to 60 Hz and peak-to-peak amplitude of 10 to 1000 μm can be applied. (B) Schematic representation of the WBV experimental design. WBV (indicated by horizontal arrows) was performed 15 min/d, 5 d/wk, on weeks 3 to 15 after MMT or sham surgery, and at the corresponding timepoints in naïve mice. Mechanical sensitivity (by von Frey filaments) and heat sensitivity (by the Hargreaves test) were evaluated a day before (baseline; BL), 2 days, and weakly on weeks 1 to 16 after MMT or sham surgery. The hind limb use (by the DWB) was assessed a day before (BL) and 2 days, 1 to 5, 9, 13, and 16 weeks after MMT or sham surgery. Locomotor activity (in the open field boxes) was assessed a day before (BL), 2 days, 1 to 3, 7, 12, and 16 weeks after MMT or sham surgery. Body weight was measured a day before (BL) and 2 days, 1 to 5, 9, 13, and 16 weeks after MMT or sham surgery. The measurements were performed before the vibration. Naïve and nonvibrated mice were examined accordingly.

The WBV was performed in a custom-made WBV device (Novotec Medical, Pforzheim, Germany), which consists of the vertically vibrating platform with attached ventilated Plexiglas cages, and can be used at adjustable vibration frequencies (15–60 Hz) and peak-to-peak amplitudes (10–1000 µm) (Fig. 1). Mice were habituated in WBV cages, without vibration. Pilot experiments optimized WBV parameters for reducing MMT-induced pain. Frequencies (15, 30, 45 Hz) were selected based on H-reflex measurements of leg soleus and extensor digitorum longus muscles. A peak acceleration of 0.3*g* was used to minimize the joint and muscular fatigue.^28^ Peak-to-peak amplitudes were calculated as amplitude (µm) = acceleration/(2π × frequency)^2^, yielding the following frequencies/amplitudes: 15 Hz/332 µm, 30 Hz/61 µm, and 45 Hz/37 µm.

Based on the pilot experiments, the WBV at 15 Hz, 332 µm, and 0.3*g* was selected for the next experiments, and was performed 15 min/d, 5 d/wk, for up to 13 weeks, on weeks 3 to 15 after MMT or sham surgery. Control animals were placed in the WBV device but not subjected to vibration (0 Hz). Naïve mice (without any surgery) were treated accordingly (Fig. 1).

### 2.4. Behavioral analysis

#### 2.4.1. Limb use

Hind limb use was assessed using a dynamic weight-bearing (DWB) device, a day before (baseline) and 2 days, 1 to 5, 9, 13, and 16 weeks after MMT or sham surgery (Fig. 1). The measurements are expressed in grams as the weight-bearing difference between ipsilateral/right and contralateral/left hind limb, when mice used either all 4 limbs (during walking or standing) or only hind limbs (during standing).^43^

#### 2.4.2. Mechanical sensitivity

Mechanical sensitivity was assessed using calibrated von Frey filaments and Plexiglas cages on a mesh stand. Mice were placed in the cages and filaments were applied to the hind paw plantar surface using the up-down method to estimate 50% withdrawal thresholds.^7^ Testing began with a 3.9-mN (0.33 *g*) filament and maximum of 6 to 9 applications were used, with the cut-off at 39.2 mN (3.59 *g*).^6,43^ Mechanical sensitivity was evaluated a day before (baseline), 2 days, and weekly on weeks 1 to 16 after MMT or sham surgery (Fig. 1).

#### 2.4.3. Heat sensitivity

Heat sensitivity was measured by the Hargreaves test in the Plexiglas chambers on a glass stand. Mice were placed in the chambers and radiant heat was applied to the hind paw plantar surface from underneath the glass floor with a high-intensity lamp bulb, and paw withdrawal latency was evaluated using an electronic timer. The withdrawal latency was averaged across 2 trials (≥10 seconds apart), with baseline latency adjusted to 10 to 12 seconds for uninjured paws, and the cut-off was 20 seconds.^43^ Heat sensitivity was assessed on the same schedule as mechanical sensitivity (Fig. 1).

#### 2.4.4. Locomotor activity

Horizontal activity was measured in the open field boxes as the distance traveled (in m) over 30 minutes.^40,43^ It was assessed a day before (baseline), 2 days, 1 to 3, 7, 12, and 16 weeks after MMT or sham surgery (Fig. 1).

#### 2.4.5. Body weight

The body weight was measured on a standard animal balance a day before (baseline) and 2 days, 1 to 5, 9, 13, and 16 weeks after MMT or sham surgery (Fig. 1).

For all tests, animals were habituated to the apparatuses before testing. Measurements were performed in the same animals, at least an hour apart (on the WBV days, before the vibration). Naïve and nonvibrated mice were examined accordingly.

### 2.5. Contrast-enhanced microcomputed tomography (µ-CT)

In additional animal groups, the WBV (15 Hz, 332 µm, 0.3*g*) was performed for 13 weeks, on weeks 3 to 15 after MMT in males and females (Fig. 1). Femur and tibia were isolated and disarticulated 16 weeks after MMT, ie, 2 days after the last WBV. Nonvibrated animals were examined accordingly. The bone pairs (femur and tibia) were formalin-fixated, ethanol-dehydrated, and incubated with shaking in 1% phosphotungstic acid in 70% ethanol as a contrast agent. For µ-CT analysis, the bone pairs were vertically positioned in custom holders and scanned using a µ-CT scanner. The 3D image stacks were reconstructed using NRecon software. A single slice was previewed before final reconstruction, and image stacks were saved for processing and segmentation (section 2.6.1).

### 2.6. Analysis of cartilage morphology

#### 2.6.1. Determination of subchondral bone and cartilage

**Figure 2.**
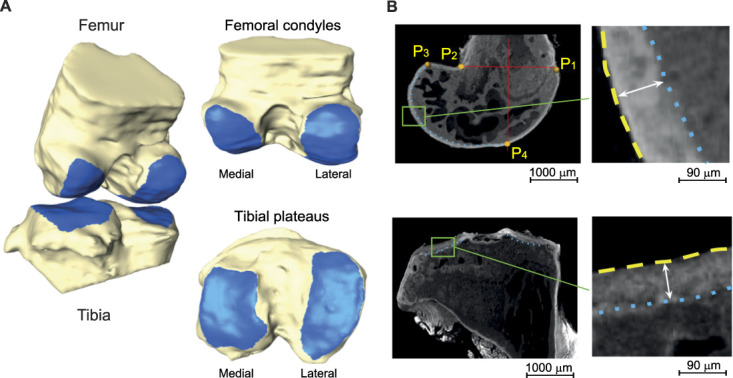
Determination of femoral and tibial subchondral bone and cartilage. (A) A 3D model of the distal femur and the proximal tibia. Left image: the femur and tibia forming the knee joint. Right images: visualization of the femoral condyles and tibial plateaus (marked blue). (B) Identification of the weight-bearing regions (WBRs) of the femur (top images) and the tibia (bottom images). Left images: In the femur, the axis was approximated perpendicular to the line connecting P1 (the most superior point of the trochlear cartilage) and P2 (defined relative to P1 on the opposite side of the femoral shaft). The WBR represents the area between P3 (the most superior point of the posterior cartilage) and P4 (the intersection of the axis and the femur margin). In the tibia, the WBRs represent entire surfaces of the plateaus. The dotted blue lines depict the subchondral bone boundaries. Right images: Magnification of the box-marked areas in the left images. The cartilage thickness represents the distance between the subchondral bone boundary (dotted blue lines) and the cartilage surface (dashed yellow lines). (A and B) Representative images of female mice subjected to WBV (15 Hz, 332 µm, 0.3*g*) were taken 16 weeks after MMT.

Femur and tibia bone and cartilage were manually segmented (https://amira.zib.de). The weight-bearing subchondral bone area of the femur was defined based on prior methodology (Fig. 2).^24^ In the sagittal view, the posterior margin of the femoral articular cartilage (P1) was identified for both condyles. The femoral mechanical axis was estimated using an anatomical landmark between P1 and P2 (a point on the opposite shaft) and computed as a line perpendicular to the one which connects points P1 and P2. The intersection of this axis and the femoral cartilage was determined (point P4). The area between points P3 (the most superior point of the posterior cartilage) and P4 defined the weight-bearing cartilage area for the femur medial and lateral condyles. The subchondral bone region of the tibial plateaus (ie, the area where cartilage is expected in a healthy knee) was manually determined and divided into the medial and the lateral plateaus for analysis.

#### 2.6.2. Computation of cartilage morphology

Femoral and tibial bone surfaces were represented as triangulated meshes.^50^ Rays were casted in normal direction to the bone surface (Fig. 2). Manual cartilage segmentations determined whether each bone surface triangle was cartilage-covered along the casted rays. The thickness (µm) was measured for cartilage-covered areas, while triangles without coverage were classified as denuded, indicating cartilage loss. The percentage of denuded regions was calculated as the ratio of denuded subchondral bone area to the total bone area covered by cartilage. Median cartilage thickness and denuded areas were computed for the medial and lateral femoral condyles individually and in combination (entire) and for the medial and lateral tibial plateaus individually and in combination (entire). The denuded areas for the entire femoral condyles and entire tibial plateaus were calculated as the mean of the medial and lateral compartment data. The cartilage thickness for the entire femoral condyles and entire tibial plateaus was calculated as the median of the medial and lateral compartment data.

### 2.7. Statistics

Two-way repeated-measures (RM) analysis of variance (ANOVA) and Sidak multiple comparisons test were used for comparing 2 groups over multiple timepoints. Two-sample comparisons used a two-tailed Student *t* test for normally distributed data or a Mann–Whitney *U* test for non-normally distributed data (Prism 6; GraphPad). Results are presented as individual data points and/or means ± SEM, with significance at *P* < 0.05.

## 3. Results

### 3.1. Whole-body vibration alleviates mechanical hypersensitivity and restores limb use in osteoarthritis

**Figure 3.**
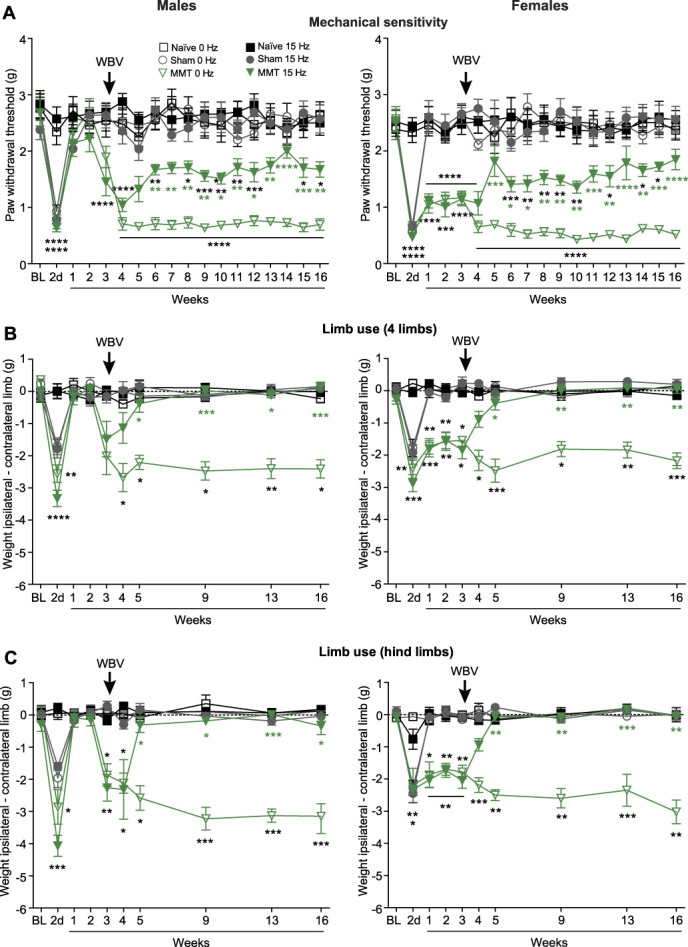
Whole-body vibration attenuates mechanical hypersensitivity and improves limb use in OA. (A) Effects of WBV on mechanical von Frey thresholds in right hind paws of naïve mice, and in right hind paws, ipsilateral to sham surgery or MMT. (B and C) Effects of WBV on the hind limb use assessed by DWB in naïve, sham-operated, and MMT mice when they used 4 limbs (walking or standing) (B) or only hind limbs (standing) (C). The limb use is expressed as the weight-bearing difference between ipsilateral/right and contralateral/left hind limbs. Negative values indicate lower weight load on operated limb. Whole-body vibration (15 Hz, 332 µm, 0.3*g*) was performed 15 min/d, 5 d/wk, on weeks 3 to 15 after MMT or sham surgery. Pain behaviors were measured before WBV. Naïve and nonvibrated (0 Hz) mice were tested accordingly. *P < 0.05, **P < 0.01, ***P < 0.001, ****P < 0.0001 vs MMT nonvibrated group (green asterisks) or vs corresponding naïve group (black asterisks); two-way RM ANOVA and Sidak test. Data are means ± SEM. N = 8 mice/group. BL, baseline (measurements before surgeries, and at the corresponding timepoint in naïve mice). 2 d, 2 days. The arrows indicate the beginning of WBV.

\We have previously shown that MMT primarily triggers mechanical hypersensitivity and reduces OA limb use, mimicking human OA.^43^ Consistently, here we observed a characteristic biphasic pattern of these pain behaviors (Fig. 3). Mechanical hypersensitivity was manifested by lower thresholds to von Frey filaments applied to hind paws (Fig. 3A), whereas poorer limb use was evidenced by reduced weight put on the OA limb when mice used either all 4 limbs (during walking or standing) (Fig. 3B) or only hind limbs (during standing) (Fig. 3C), as assessed by DWB. The biphasic pattern included an acute postoperative phase (peaking on day 2) and a chronic OA phase lasting over 3 months (weeks 3–16), separated by a remission in the intermediate phase (weeks 1–2) in males. Females displayed a less pronounced biphasic response, as they did not fully recover in the intermediate phase (Fig. 3). The acute postoperative pain was supported by mechanical hypersensitivity and poorer limb use also observed after sham surgery. By contrast, in the chronic phase, the MMT-induced both pain behaviors apparently relate to the OA pain, as sham surgery did not induce these effects (Fig. 3). These findings are consistent with previous studies.^16,21,43^

We then examined these pain behaviors in pilot experiments to identify the most effective WBV parameters for mitigating pain, using a WBV device (section 2.3, Fig. 1 and see Movie S1, supplemental digital content, http://links.lww.com/PR9/A403). These experiments indicated the improvement of mechanical hypersensitivity and limb use in both males (see Fig. S1A-C, supplemental digital content, http://links.lww.com/PR9/A403) and females (see Fig. S2A-C, supplemental digital content, http://links.lww.com/PR9/A403) by WBV at 15 Hz, 332 µm, and 0.3*g*. Consequently, for the subsequent experiments, we used WBV at these parameters, and performed it 15 min/d, 5 d/wk, for up to 13 weeks, on weeks 3 to 15 after MMT or sham surgery, in both sexes. Naïve mice (without any surgery) were treated accordingly. We found that this WBV treatment consistently, but partially ameliorated mechanical hypersensitivity, depicted by a statistically significant difference compared with naïve mice exposed to WBV (Fig. 3A). Interestingly, WBV fully restored the OA limb use (to the values of naïve and sham-operated mice exposed to WBV, and the baseline values measured before surgeries), when animals used either all 4 limbs (Fig. 3B) or only hind limbs (Fig. 3C). The pain improvement appeared 2 weeks after WBV initiation (ie, 5 weeks after MMT) and persisted for up to 4 months after MMT (16 weeks) in both sexes (Figs. 3A–C). The control treatment (placing animals in the WBV apparatus without inducing the vibration) had no effects. There were also no changes in sham-operated and naïve animals after WBV as compared with control, not vibrated mice (Figs. 3A–C).

### 3.2. Whole-body vibration does not modify heat sensitivity, locomotor activity, and body weight in osteoarthritis

**Figure 4.**
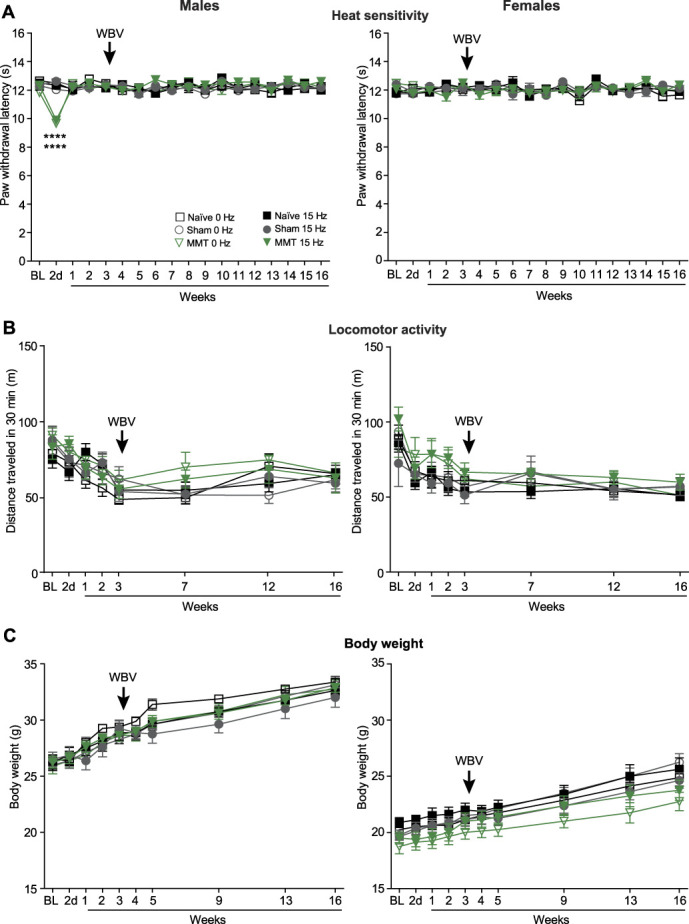
WBV does not modify heat sensitivity, locomotor activity, and body weight in OA. (A) Effects of WBV on heat latencies in right hind paws of naïve mice, and in right hind paws, ipsilateral to sham surgery or MMT, measured using the Hargreaves test. (B) Effects of WBV on locomotor activity measured as a distance (in m) travelled during 30 minutes in open field boxes. (C) Effects of WBV on body weight. All effects were evaluated in naïve, sham-operated, and MMT mice with or without WBV. The WBV (15 Hz, 332 µm, 0.3*g*) was performed 15 min/d, 5 d/wk, on weeks 3 to 15 after MMT or sham surgery. The effects were measured before the WBV. Naïve and nonvibrated (0 Hz) mice were tested accordingly. The WBV did not affect any behavior and body weight, *P* > 0.05, MMT vibrated vs MMT nonvibrated (0 Hz) groups; 2-way RM ANOVA. ****P < 0.0001 vs matching naïve groups; two-way RM ANOVA and Sidak test. Data are means ± SEM. N = 8 mice/group. BL, baseline (measurements before MMT or sham surgery, and at the corresponding timepoint in naïve mice). 2 d, 2 days. The arrows indicate the beginning of WBV.

To validate the importance of WBV in alleviating OA-related pain, we also assessed the WBV effects on other behaviors (Fig. 4). Besides a transient, postoperative heat hypersensitivity assessed by the Hargreaves test (manifested by shorter withdrawal latency to heat applied to hind paws; day 2) in males, the MMT did not induce heat pain in the chronic OA phase in either sex (Fig. 4A). The MMT also did not modify the locomotor activity measured in the open field test (Fig. 4B) and the body weight (Fig. 4C), compared with sham-operated and naïve mice, in both sexes. Importantly, none of these behaviors were altered by WBV (15 Hz, 332 µm, 0.3*g*), and there were no changes in sham-operated and naïve animals (Fig. 4). The heat sensitivity and locomotor activity were also unaltered by WBV at the other parameters (35 Hz/61 µm, 45 Hz/37 µm) (see Figs. S1D and E, Fig. S2D and E, supplemental digital content, http://links.lww.com/PR9/A403). These data suggest that WBV does not alter general behavior, but selectively alleviates OA-related mechanical pain and ameliorates limb dysfunction, which are the predominant features of clinical OA.^11,25^

### 3.3. Whole-body vibration reduces the osteoarthritis-induced cartilage degeneration

**Figure 5.**
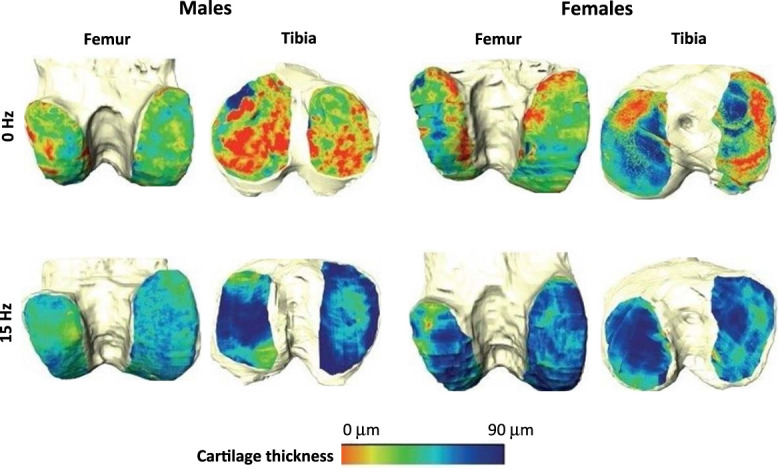
Representative µ-CT heat maps of femoral and tibial subchondral bone and cartilage of OA mice without or with WBV. The images show femoral condyles and tibial plateaus of males (left images) and females (right images) at week 16 after MMT, ie, 2 days after the last WBV (15 Hz, 332 µm, 0.3*g*) (bottom images) and at corresponding timepoint in nonvibrated (0 Hz) mice (top images). The WBV was performed 15 min/d, 5 d/wk, on weeks 3 to 15 after MMT. Nonvibrated mice were tested accordingly. Cartilage thickness is visually represented on a color scale with red denoting thinner regions (toward 0 μm) and the blue indicating thicker areas (toward 90 μm).

**Figure 6.**
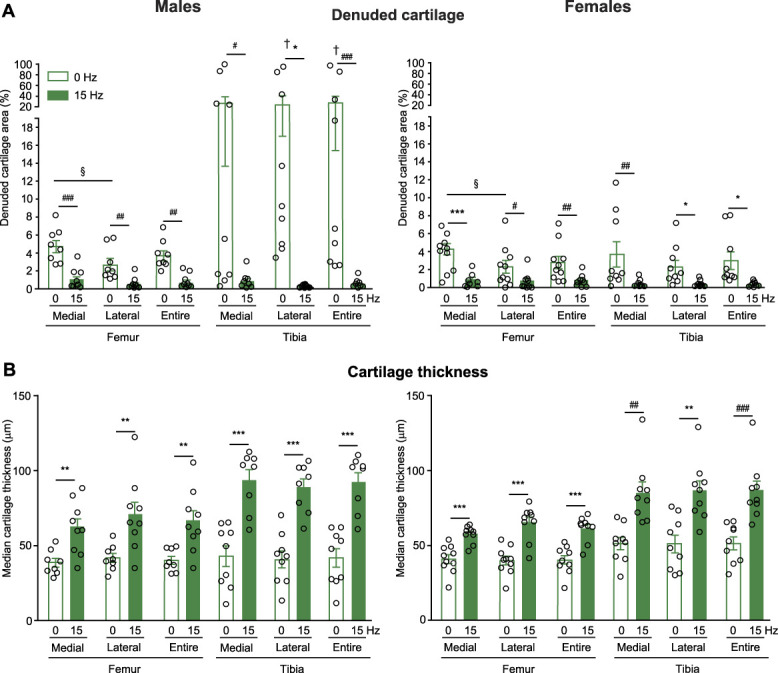
WBV reduces cartilage degeneration in OA. (A–B) Effects of WBV on cartilage denudation expressed as the percentage of subchondral bone denuded of cartilage (A), and cartilage thickness expressed in µm (B) in MMT knees. The cartilage parameters are shown for femur medial and lateral condyles individually and in combination (entire), and for tibia medial and lateral plateaus individually and in combination (entire). The WBV (15 Hz, 332 µm, 0.3*g*) was performed 15 min/d, 5 d/wk, on weeks 3 to 15 after MMT. The femur and tibia were isolated at week 16 after MMT, ie, 2 days after the last WBV, and analyzed by µ-CT. The nonvibrated (0 Hz) mice were examined accordingly. *P < 0.05, **P < 0.01, ***P < 0.001 (*t* test), #*P* < 0.05, ##*P* < 0.01, ###*P* < 0.001 (Mann–Whitney *U* test) vs corresponding nonvibrated group of the same sex. §*P* < 0.05 (Mann*–*Whitney *U* test), medial vs lateral femur of the nonvibrated mice of the same sex. †*P* < 0.05 (Mann–Whitney *U* test) vs females in the corresponding groups. Data are individual data points and means ± SEM. N = 8 to 10/group.

To evaluate the effect of WBV on cartilage morphology, the WBV (15 Hz, 332 µm, 0.3*g*) was performed for 13 weeks, on weeks 3 to 15 after MMT in both sexes, consistent with behavioral experiments. Femur and tibia were collected at week 16 after MMT, ie, 2 days after the last WBV. The animals not subjected to WBV were examined accordingly. In the isolated bones, we segmented the µ-CT data and analyzed cartilage degeneration for the weight-bearing femoral and tibial subchondral bone areas (section 2.6). Cartilage thickness was computed for both bones, and visualized as heat maps, with a color scale ranging from red (indicating thinner regions) to blue (indicating thicker areas) (Fig. 5). We found that MMT resulted in cartilage degeneration, depicted as red regions (Fig. 5), and manifested by a substantial percentage of subchondral bone denuded of cartilage in femur and tibia of control, nonvibrated males and females (Fig. 6). The percentage of denuded cartilage in the lateral tibial plateau was higher in males than in females; a corresponding result was found when the denuded cartilage was computed for both lateral and medial plateaus in combination (entire; Fig. 6A). However, no significant sex differences were found in the medial tibial plateau or the femur. In the femur, there was a higher percentage of denuded cartilage in the medial condyle compared with the lateral condyle, in both sexes (Fig. 6A). Importantly, WBV substantially reduced cartilage degeneration, as indicated by increased areas of blue regions on heat maps (Fig. 5) and demonstrated by a substantially diminished percentage of denuded cartilage in femur and tibia of males and females (Fig. 6A). In addition, WBV increased the cartilage thickness in both bones of both sexes (Fig. 6B).

## 4. Discussion

Our study revealed that WBV considerably ameliorated pain behaviors and reduced cartilage degeneration in male and female mice with knee OA. These findings are important because: (1) WBV is a nonstrenuous therapy suitable for elderly patients with OA who may be unable to engage in intensive exercise, (2) pain is a debilitating OA symptom for which effective interventions are warranted, and (3) cartilage erosion drives OA progression, necessitating therapies that halt or reverse it.

Clinical studies showed that WBV combined with strengthening exercises diminishes pain and improves physical function in patients with musculoskeletal disorders, including low back pain and knee OA.^2,31,36,39,46^ While an earlier review presented inconclusive evidence,^47^ latter systematic reviews and meta-analyses of randomized, controlled clinical trials confirmed that adding WBV to strengthening exercises reduces pain more effectively than exercises alone in patients with knee OA,^34,51^ without exerting adverse events.^34^ However, the impact of WBV alone remains underexplored.^51^ Given this gap, preclinical models offer valuable insights into the effects of sole WBV on OA.

To ensure stringent conditions, we have codesigned a custom-made mouse WBV device with precise vibration parameters' control, and compartments allowing for animal free movements to avoid restrain stress, which could confound the results. Based on pilot experiments, we selected WBV at 15 Hz, 332 µm, and 0.3*g*, and applied it 15 min/d, 5 d/wk, for up to 13 weeks, on weeks 3 to 15 after MMT. We began WBV at 3 weeks after MMT to examine the effects of WBV on pain in the chronic OA phase, lasting 3 to 16 weeks after MMT. This treatment substantially diminished MMT-induced mechanical hypersensitivity and restored MMT-induced limb use impairment compared with naïve and sham-operated animals, placed in the WBV device, but not exposed to vibration, in both males and females. Whereas mechanical hypersensitivity was partially reversed by WBV, the limb use was completely restored, both during walking and standing. Mechanical hypersensitivity, assessed using von Frey filaments on the OA limb's hind paw, reflects sensitivity to punctate tactile stimuli and referred pain (ie, pain in body parts remote to the injury). Limb use, measured by DWB (in which animals can freely move), indicates spontaneous and/or movement-evoked pain. Both types of pain are common in patients with OA.^11,25,43^ Apparently, despite incomplete resolution of mechanical tactile hypersensitivity, WBV enabled animals to bear weight on their OA limb comparably with animals without OA. These beneficial effects emerged 2 weeks after WBV initiation and were measured every week, 2 days after preceding WBV session, up to 4 months after MMT. These findings indicate a gradual and sustained pain relief, persisting at least 2 days without WBV application. Other studies using the anterior cruciate ligament transection (ACLT) OA model in rats reported worsened pain behaviors (assessed by CatWalk gait analysis) after WBV at 35 Hz and 0.3*g*.^33^ In rabbits with ACLT, the WBV was detrimental at 40 Hz and 0.3*g*, while lower frequencies 10 to 20 Hz and 0.3*g* attenuated pain behaviors (assessed by incapacitance test).^18^ Neither WBV amplitude nor animal sex was reported in those studies.^18,33^ Notably, our pilot experiments showed that in contrast to 15 Hz, WBV at 35 Hz and 45 Hz was ineffective in reducing mechanical hypersensitivity or improving OA limb use. Hence, the effects of WBV on OA pain can depend on vibration parameters. Considering animal sex, we observed no differences between males and females in mechanical hypersensitivity and impaired OA limb use during the chronic OA phase, consistent with our earlier study,^43^ and WBV improved both pain behaviors to a similar degree in both sexes. Heat sensitivity, general locomotor activity, or animal body weight (which were unaltered by MMT) were not modified by WBV. Together, our findings demonstrate that WBV (15 Hz, 332 µm, 0.3*g*) selectively alleviates OA-related pain behaviors without causing adverse effects, irrespective of animal sex.

Furthermore, WBV reduced MMT-induced knee cartilage degeneration, assessed by µ-CT, in both sexes. Cartilage damage was manifested by a considerable percentage of subchondral bone denuded of cartilage in femur and tibia. The denudation in the lateral tibial plateau was stronger in males than in females, consistent with previous findings demonstrating more severe cartilage damage in males.^5,15,23,43^ The underlying mechanisms include hormonal influences (estrogen-induced chondroprotective effects, testosterone-induced exacerbation of cartilage degeneration), higher body weight, and greater inflammation (synovitis, activity of proteases degrading cartilage, proinflammatory transcriptomes) in males.^23^ Notably, WBV substantially diminished the bone denudation and increased cartilage thickness in medial and lateral compartments of both bones in males and females. To the best of our knowledge, no studies have compared WBV effects on either OA pain or articular cartilage between sexes/genders. Following our pain behavior protocol, we started WBV 3 weeks after MMT, when chronic OA phase had begun, and performed µ-CT at week 16 after MMT, 2 days after the last WBV. These data suggest that WBV mitigated already established cartilage damage, and these protective effects persisted after WBV cessation. These findings are notable given the cartilage's limited regenerative capacity in OA.^3,37^ WBV effects on cartilage may depend on animal strain/genotype and WBV parameters. Thus, 45-Hz and 0.3*g* WBV enhanced cartilage degeneration in naïve CD1 mice,^26,27^ but not in naïve C57BL/6 mice.^19^ Similarly, in ACLT-induced OA in rats or rabbits, WBV increased cartilage damage at 35 to 40 Hz, but decreased it at 20 Hz (0.3*g*).^18,33^ These findings are based on histological cartilage examination in males,^19,26,27^ while other studies did not state the animal sex.^18,33^ Our data show that WBV (15 Hz, 332 µm, 0.3*g*) reduced cartilage degeneration in OA-affected limbs in both male and female mice. Notably, a clinical trial in healthy young men reported that WBV (20 Hz, 2–4 mm) prevented immobilization-induced knee cartilage thinning,^22^ but studies in patients with OA are needed.

The action of WBV relies upon the transmission of vibrations to the body from the vibrating device.^32,35^ Cartilage chondrocytes respond to vibratory stimuli,^32^ and WBV may promote cartilage regeneration by enhancing synthesis of EM components such as proteoglycans^42^ and reducing matrix-degrading enzyme expression.^30^ Pain relief from WBV is believed to result from improved muscle function and/or direct inhibition of nociceptive neurons.^35,45^ In addition, WBV may improve weight distribution, reducing mechanical stress on cartilage and supporting repair. However, the precise cellular and molecular mechanisms remain to be elucidated. Future studies might examine nociceptive fiber density, EM components, and endogenous opioid systems.

A limitation of our study may be the absence of naïve and sham-operated animals in the µ-CT assessment of cartilage. Consequently, we cannot determine how closely the cartilage in WBV-treated MMT animals resembles that of naïve and sham-operated animals. Importantly, our extensive in vivo testing revealed that these animals did not show changes in any behavioral parameters, including mechanical and heat sensitivity, limb use, locomotion, or body weight during the phase corresponding to the chronic MMT phase (weeks 3–16), and did not display joint histopathology demonstrated in our earlier study.^43^ Therefore, by showing that WBV diminished MMT-induced cartilage degeneration, our primary objective to examine WBV effects on OA-induced cartilage damage has been met. Nevertheless, inclusion of naïve and sham-operated controls in subsequent imaging analyses will be valuable, as pain and cartilage pathology are not necessarily concordant in OA, and behavioural outcomes alone cannot predict the cartilage status. In addition, future studies might address: (1) WBV efficacy when initiated at later OA stages (beyond 3 weeks), as clinical OA can be long-lasting; (2) maximal duration of beneficial effects after WBV cessation, (3) outcomes in older animals, given the OA prevalence with age, and (4) WBV effects on other joint pathology features such as synovitis, subchondral bone changes, and osteophyte formation.

Our findings have important implications, as they demonstrate that WBV alone can alleviate pain and reduce cartilage damage in a clinically relevant knee OA model,^43^ supporting testing of sole WBV in patients with OA. WBV conditions will need to be optimized for humans, as the vibration characteristics depend on body position (up-right standing in humans vs four leg walking in mice) and the type of vibratory platforms used for humans (side-to-side or simultaneous vertical, or multidirectional oscillations).^32,35,45^ In addition, many clinical studies are of poor quality due to underpowered designs, inappropriate statistical methods, and unspecified vibration characteristics,^34,47,51^ making it crucial to establish robust WBV protocols for future trials.

## Disclosures

The authors have no conflicts of interest to declare.

## Supplemental digital content

Supplemental digital content associated with this article can be found online at http://links.lww.com/PR9/A403.
